# An Ultra-Low Power Wireless Sensor Network for Bicycle Torque Performance Measurements

**DOI:** 10.3390/s150511741

**Published:** 2015-05-21

**Authors:** Sadik K. Gharghan, Rosdiadee Nordin, Mahamod Ismail

**Affiliations:** 1 Department of Electrical, Electronic and Systems Engineering, Faculty of Engineering and Built Environment, Universiti Kebangsaan Malaysia, UKM Bangi, Selangor 43600, Malaysia; E-Mails: adee@ukm.edu.my (R.N.); mahamod@eng.ukm.my (M.I.); 2 College of Electrical and Electronic Engineering Techniques, Foundation of Technical Education, Baghdad 10062, Iraq

**Keywords:** ANT, bicycle torque, energy-efficient, track cycling, wireless sensor network, ZigBee

## Abstract

In this paper, we propose an energy-efficient transmission technique known as the *sleep/wake* algorithm for a bicycle torque sensor node. This paper aims to highlight the trade-off between energy efficiency and the communication range between the cyclist and coach. Two experiments were conducted. The first experiment utilised the Zigbee protocol (XBee S2), and the second experiment used the Advanced and Adaptive Network Technology (ANT) protocol based on the Nordic nRF24L01 radio transceiver chip. The current consumption of ANT was measured, simulated and compared with a torque sensor node that uses the XBee S2 protocol. In addition, an analytical model was derived to correlate the sensor node average current consumption with a crank arm cadence. The sensor node achieved 98% power savings for ANT relative to ZigBee when they were compared alone, and the power savings amounted to 30% when all components of the sensor node are considered. The achievable communication range was 65 and 50 m for ZigBee and ANT, respectively, during measurement on an outdoor cycling track (*i.e.*, velodrome). The conclusions indicate that the ANT protocol is more suitable for use in a torque sensor node when power consumption is a crucial demand, whereas the ZigBee protocol is more convenient in ensuring data communication between cyclist and coach.

## 1. Introduction

The torque generated by a cyclist during crank arm rotation is considered to be the most critical performance index for competitive cyclists. Thus, the evaluation of the athlete’s power performance has become of primary interest for the cyclists and coaches [1]. During track competition, cyclists aim to produce a maximal power output over the entire cycling ride duration, where the power delivered by the crank arms is converted into bicycle speed [2]. The cyclist’s power can be measured by using a device known as a power meter, which is based on the pedal torque or crank arm torque measurements. Due to technological developments in recent years, bicycle power meters have become part of the training equipment of professional cyclists. However, such devices have the following shortcomings: (I) the bicycle torque cannot be monitored by the coach in real time, as there is no real-time data transmission between the cyclist and the coach; and (II) they have a short communication range. Currently, the torque sensor node (which is installed on the bicycle crank arm) is able to transmit the bicycle torque data to the power control device (which is installed on the cyclist handlebar); and (III) the battery of the torque sensor node cannot be replaced or recharged by the user because the device is packaged in a *black box*. Hence, the users must send the power meter back to the manufacturer to replace the battery. 

Due to these shortcomings, a proposed wireless sensor network (WSN) system was designed and implemented that is capable of monitoring and transferring bicycle crank arm torque data. The proposed torque measurement system consists of three nodes: (I) the sensor node; (II) the router node; and (III) the coordinator node. This system depends on the right crank arm torque measurement, using four strain gauges, where the strain gauges are glued to this one and connected as full Wheatstone bridge circuit. Left crank arm torque measurement is not implemented in this study to significantly reduce the energy consumption, to miniaturize the size and weight, to reduce the aerodynamic resistance, and to reduce the cost. In this case, a total torque estimation can be obtained by multiplying the measured torque or power in the right crank arm by two. This approach was adopted because of the assumption that the torque or power produced by both limbs is nearly identical [3], similar to reference [4]. Consumer-ready systems, such as Ergomo Pro (Ep) and Garmin Vector (Vector S), follow this approach. Vector S is a “single-sensing system that measures the forces on the left pedal to approximate total power” [5]. This procedure was proven in [6], where Torque_right_ = 0.988 Torque_left_ and Power_right_ = 1.002 Power_left_.

The measured data of the torque produced by the right crank arm are transmitted wirelessly to the coordinator node (*i.e.*, coach) based on the wireless protocol through the router node. However, there are several criteria focusing on sensor node power consumption that can be taken into account to overcome these shortcomings based on the layers used in wireless protocols as follows:

(I) Application layer: load division technique to avoid intensive computation in the sensor node. The task can be performed by the coordinator node (*i.e.*, the torque is measured in the sensor node, and the bicycle power calculated in the coordinator node, where power = torque × cadence × (2π/60)). The bicycle cadence was measured in our previous work, which presented in [7].
(II) Transport layer: no resending of packet losses because the data transmitted from the sensor node are updated every crank arm rotation, in seconds or milliseconds.
(III) Network layer: the transfer of data between a bicycle and coach established through one-hop routing, which consumes less energy in the network.
(IV) Data link layer: the traditional wireless sensor network consists of a sensor node, the router node, and the coordinator node. When a packet error occurs, the router node automatically requests the re-send of the packet loss from the sensor node utilising the automatic repeat request (ARQ) and forward error correction (FEC) techniques. That method is performed without the knowledge of the coordinator node, which reduces the retransmission overhead. In our application (*i.e.*, bicycle torque monitoring), the ARQ method is not used because the data are updated every crank arm rotation and the data error correction is transmitted at the end of the data packet. Hence, the power consumption is reduced in the router and coordinator nodes.
(V) Media Access Control (MAC) Layer: sleep scheduling can be performed to bring the Radio Frequency (RF) module to sleep mode for a particular time depending on the duty cycle of the bicycle crank arm rotation. In most applications, the RF module of the sensor node spends most of its time in sleep mode, waking up based on a sensing event or scheduling scheme to transmit data and then returning to sleep again. In our application, the RF module wakes up for each rotation, transmits torque data quickly in a fraction of time, and returns to sleep mode again. This strategy can significantly reduce the energy consumption. However, in our current application, the sensor node cannot sleep for a very long time because the torque data are transmitted every crank arm rotation in seconds or milliseconds. Therefore, a new proposed *sleep/wake* algorithm was designed and implemented to reduce the power consumption, achieve energy savings, and prolong the battery life of the torque sensor node.
(VI) Physical layer: a suitable hardware design and interface with a proper microcontroller to dramatically reduce the power consumption and prolong the battery life.

In this study, to select the most appropriate wireless protocol in terms of energy savings and communication range, two experiments were performed. The first experiment was based on the XBee S2 wireless protocol, whereas the second was based on ANT. The use of the XBee S2 and ANT wireless standards addresses the requirements of low power consumption, low cost and weight, small size [8,9], suitable communication range, and free license Industrial, Scientific, and Medical (ISM) band 2.4 GHz wireless application [10]. An emerging wireless technology is Bluetooth Smart, which was previously called Bluetooth Low Energy (BLE) [11]; it is characterized by low power consumption, high data rate, and small size [12]. BLE wireless technology allows new low-cost Bluetooth Smart devices to be operated for months or years on small, coin-cell batteries. However, BLE has a short communication range [13,14,15], and its performance remains to be proven since BLE is a new technology (introduced in July 2010) although several manufacturers have announced their support for this wireless technology [16]. Considering the aforementioned reasons, BLE was not considered in this study.

The contributions of this paper are (I) the design and implementation of a wireless bicycle torque measurement system based WSN that ensures the data communication between a bicycle and the coach on a track cycling field (*i.e.*, velodrome) over 65 m (which represents the maximum length of the cycling track of the adopted velodrome); (II) the sensor node current consumption was significantly reduced using the proposed *sleep/wake* algorithm; (III) the formulation of mathematical derivations and an analytical model that accurately represent the current consumption and battery life of the sensor node at different pedalling rates; (IV) the power consumption of the WSN was reduced by selecting the most appropriate wireless protocol based on experimental measurements; and (V) torque data aggregation is performed by transmitting the average value of 60 samples in the power stroke region. Hence, the sensor node does not need to send all of the sensed data, but rather the average data are transmitted one time every crank arm rotation. Thus, a considerable amount of energy can be saved by reducing the RF communication.

## 2. State of the Art

In track cycling, assessing the cyclist performance and training organisation can be performed using instrumented devices and cycling sports trainers. For this purpose, torque measurement has been provided by manufacturers as commercial products and has also been the subject of research. Some of the previous studies, which will be discussed in this section, measured bicycle power. The measurements were achieved based on torque or force measurements using strain gauge or force sensors. One study [17] presented a communication system that ensures the data transfer between cyclists and the coach for 50 m in real time. The system was adopted based on the commercial Ergomo™ power meter and MicaZ motes to monitor the bicycle power. A limitation of that study is the battery lifetime of up to 20 h before it has to be recharged again. Drouet *et al.* [18] measured the cyclist power based on the applied force of the two components *Fz* and *Fx* on the pedals. However, the data acquisition system (including the backpack carried by the cyclist) is restricted by wired connections between the pedals and encoder as well as its weight, which is 1.2 kg. A modification of the Schoberer Rad Messtechnik (SRM) system was performed by Barratt [19] to overcome the cyclist power measurement limitation at the beginning of the cycle. The hardware modification or torque box connects the power control with the power meter using a wire connection. The torque box is installed under the cyclist’s post seat; it is too large and adds additional weight to the bicycle, especially when it is used in competition, and it also adds aerodynamic resistance. Dorel *et al.* [20] used an instrumented pedal to measure the normal and tangential forces on both the left and right pedals. The data were monitored wirelessly. However, the data acquisition system, which is carried by the cyclist in his backpack, was heavy, thereby adding extra weight onto the cyclist when the system is used in training or competition.

A proposed design by Bibbo *et al.* [21] was used to measure the applied force on the bicycle pedal. The measured data were transmitted to a PDA device based on an ad hoc design. As is well known, PDA devices using Bluetooth technology to transfer data are characterised by a short communication range and high energy consumption. A commercial product, the THUN X-CELL RT pedal sensor, was used by Spagnol *et al.* [6] to measure the torque applied to the left pedal. An algorithm was proposed to estimate the right torque to determine the whole cyclist torque. The experimental results show a high correlation between the torque or power of the right and left pedalling revolutions. However, the system used a commercial product that was not developed by the author’s design. Moreover, the method of torque monitoring was not provided in the research. Vanwalleghem *et al.* [4] proposed a system to measure the pedal power based on the applied force on the right pedal. The total power was obtained by multiplying the right pedal power by two. The pedal power was transmitted via Bluetooth. However, classic Bluetooth technology has a low transmission range and consumes more power than ZigBee and ANT technologies. Finally, Bini and Carpes [2] measured the applied normal force component *Fy* on the bicycle pedal. However, this design was not integrated with a wireless system.

The main challenges in WSNs involve reducing the power consumption of the RF communication module and data processing unit. This reduction is a challenge because the WSNs are limited energy sources, and most of their energy is dissipated by transmitting and receiving the data. Therefore, an efficient energy strategy must be proposed that aims to reduce the torque sensor node power consumption, especially when the bike is on the track, where the batteries of the sensor nodes cannot be replaced or recharged instantly. An efficient hardware must also be selected. In this paper, two types of wireless protocol were used and investigated in terms of current consumption, XBee S2 and ANT protocols. Consequently, the following two wireless technologies will be highlighted to consider the best in terms of power saving.

In WSNs, the ZigBee protocol is commonly used in different applications. ZigBee is a wireless standard that operates in the 868 MHz, 915MHz, and 2.4 GHz ISM band [22,23]. In this paper, ZigBee (XBee S2) 2.4 GHz was adopted. This protocol is targeted for low power consumption, low data transmission rate, low cost, high reliability, and high safety, compatibility with one another [24], and a relatively long communication range [25]. ZigBee was developed for reliable wireless control and network monitoring [26], and it is therefore suitable for the aims of this paper.

The ANT wireless protocol is a proprietary communication protocol for ultra-low power consumption widely used in wireless body area networks (WBAN). The protocol was designed by Dynastream Innovations, Inc. and Texas Instruments [27]. It operates in the 2.4 GHz ISM band using Gaussian frequency shift keying (GFSK) modulation [28]. It is characterised by ultra-low power, low cost, small size and working at rates of 250 kbps, 1 Mbps, and 2 Mbps with three different modes of communication: (I) acknowledged; (II) broadcast and (III) burst. ANT employs a time division multiple access (TDMA) technique, where communication happens within predetermined time periods [29], which will eliminate collisions and improve power efficiency and channel utilisation [30]. However, ANT application for transmitting and receiving data for several meters distance has not yet been adopted [28]. Therefore, the current work will explore transferring torque data between two ANT modules. The ANT module was able to transfer the torque data for 50 m between the cyclist and coach. Compared to most common wireless technologies such as Bluetooth low energy and Zigbee, the ANT protocol has the following advantages [28,29,31].

- The transmitted power can be adapted to four levels (*i.e.*, for nRF24L01 module): 0 dBm, −6 dBm, −12 dBm, and −18 dBm [32]. The corresponding current consumptions are 11.3 mA, 9 mA, 7.5 mA, and 7 mA.
- The current consumption is 11.83 mA in reception mode, 26 µA in standby mode, and 900 nA in power-down mode [33].
- Configurable data rate (250 kbps, 1 Mbps, and 2 Mbps).
- Configurable packet size.
- Possibility to connect to a low-cost 4-to-8 bit microcontroller.
- It has frequency agility to ensure the robustness of communication between ANT devices in the case of channel degradation due to Wi-Fi, ZigBee, and Bluetooth interference [34].

Through these reviews, we found several gaps and limitations related to a wireless bicycle torque measurement that pose challenges for track cycling applications. None of the previous research works address torque sensor nodes in terms of power consumption or communication range. In addition, none of the commercial products, such as SRM and Garmin Vector, ensure real-time data transfer between the coach and cyclists while on the track field (*i.e.*, velodrome). Most of these consumer-ready devices do not monitor the torque measurement since the applications is limited to power measurement, while power measurement can be done through an extension of a torque mathematical formula, which presented earlier in the introduction section. These limitations motivated us to develop a prototype of a wireless bicycle torque measurement system that ensures torque and power monitoring, low power use and allows real-time data transfer between the cyclist and coach utilising low-power wireless technology.

## 3. Conceptual Framework

**Figure 1:**
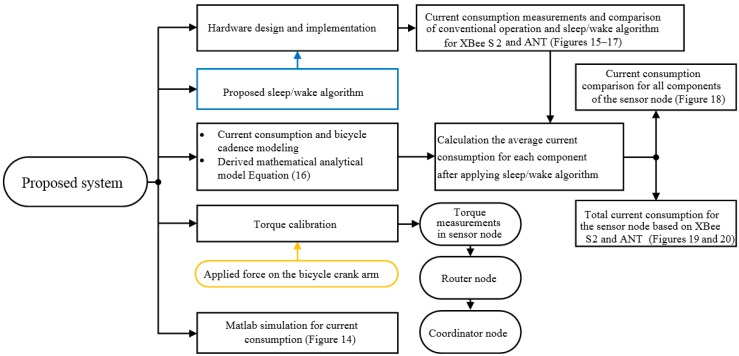
Conceptual research framework for the bicycle sensor torque measurement system.

The conceptual framework of our research is shown in Figure 1. It can be classified into five phases as follows:

1- The torque measurement system was designed and implemented using hardware components such as XBee S2, ANT wireless protocol, microcontroller Atmega 328p, and instrumentation amplifier AD623, which consume little power.
2- A proposed *sleep/wake* algorithm was implemented, which aims to reduce the current consumption of the hardware components of the torque sensor node.
3- The current and time use of the XBee S2 and ANT wireless protocols were modelled based on active and sleep current measurements and time calculations. Consequently, the comparison between these two wireless protocols was achieved in terms of power consumption to prove the algorithm performance by investigating the power savings. In addition, the active and sleep current consumption for each component in the sensor node was measured. The average current consumption of each component and the total current consumption of the sensor node while applying the *sleep/wake* algorithm was calculated based on the measured current consumption and a derived analytical model. As a result, the power saving and battery lifetime of the torque sensor node were estimated.
4- The static torque calibration process was performed based on the strain gauge sensors, amplifier circuit and applied force to the bicycle crank arm. The calibration process is necessary to find a relationship between the applied force on the crank arm and strain gauge output voltage to measure the torque produced in the crank arm during cyclist pedalling. The torque value is transmitted from the sensor node every crank arm rotation to the coordinator node via a router node.
5- Finally, the current consumption was investigated relative to the crank arm angle using a MATLAB simulation. Consequently, the current use was evaluated for each component in the sensor node at 0°, 180° and 360° crank arm angles, where these angles represent the magnetic sensor locations on the bicycle chainring.

## 4. Design Description

**Figure 2:**
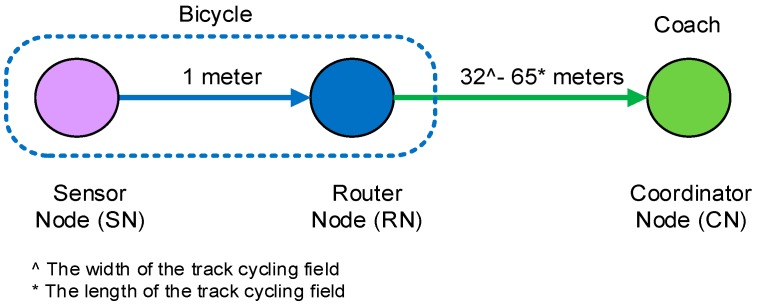
Bicycle network topology: data transmitted through one router.

The bicycle network topology consists of a three wireless nodes, which are the sensor node (SN), the router node (RN), and the coordinator node (RN) as shown in Figure 2. Both of the SN and RN are installed on the bicycle, with the SN installed onto a bicycle crank arm and the RN installed under the rider post seat to reduce the aerodynamic resistance of the bike. The CN is located in the coach’s location to receive the transmitted torque data. The SN acquires and processes the torque data and sends it to the CN through the RN.

**Figure 3:**
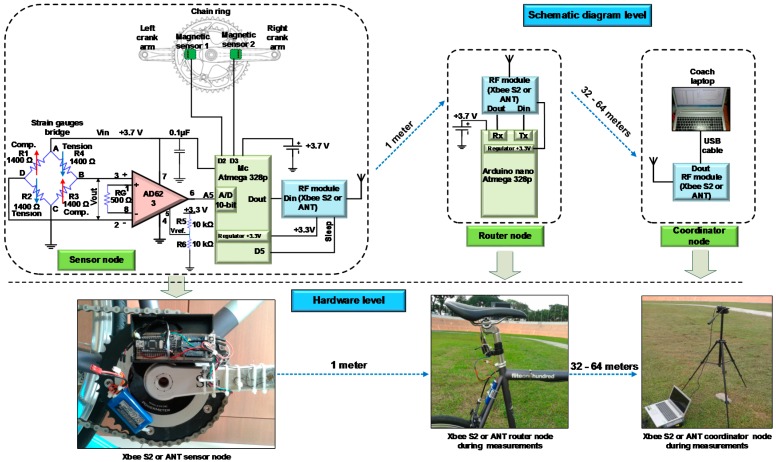
Schematic and hardware diagram of the whole WSN.

The schematic and hardware diagram of the whole system is shown in Figure 3. The SN includes: a permanent magnet, two magnetic sensors, a four strain gauge transducer forming a Wheatstone bridge installed on the right crank arm of the bicycle, instrumentation amplifier AD623, stand-alone microcontroller Atmega 328p, RF wireless module (*i.e.*, ZigBee or ANT), and LiPo rechargeable battery 3.7 V/1000 mAh. The RN consists of the RF wireless module and Atmega 328p microcontroller. The RN used compulsorily in this network due to the SN is not able to transfer the data directly to the CN (*i.e.*, coach location). This conclusion is drawn based on several experiments to send data directly from the SN to CN. However, all attempts were unsuccessful due to the low height of the SN, as it installed on the bicycle crank arm (that normally has a low height). The CN consists of the RF wireless module and laptop to allow the coach to monitor the cyclist’s torque or power based on the graphical user interface (GUI). The following subsections will describe in details the mechanical and electrical design and implementation of the WSN torque measurement system.

### 4.1. Strain Gauges Sensor Installation

A strain gauge is a physical device that can be used to measure the strain of the surface, where the strain increases when the distance from the applied force point increases [35]. This gauge is considered as a sensor or transducer that can be used to convert mechanical change into an electronic signal. It is commonly utilized to measure and record the torque applied to a rotating system (*i.e.*, rotor, bicycle crank, crankshaft, gearbox, or engine), force, displacement, weight, and pressure. The general idea of the strain gauge is if a gauge resistance is held under strain or compression, resistance length *L* becomes slightly longer or shorter, and its cross-sectional area *A* increases or decreases. Therefore, resistance *R* of the strain gauge can be calculated from Equation (1) [36]:

(1) $$R=\text{ρ}\frac{L}{A}$$

where ρ is the strain gauge wire resistivity.

**Figure 4:**
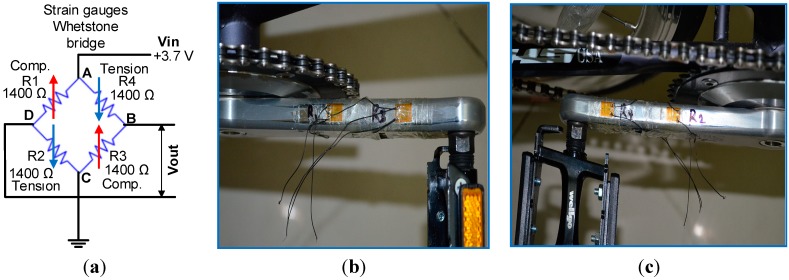
Strain gauge: (**a**) Wheatstone bridge; (**b**) installation on the top side of the crankset, and (**c**) installation on the bottom side of the crankset.

The equation above shows that a relationship exists between resistance value and length of the resistor. When the length changes, the resistance value also changes. When the strain gauge zero point is affected by temperature change, an error in measurement occurs. A Wheatstone bridge is helpful in overcoming this error and provides an accurate measurement. Four strain gauges (*R*_1_, *R*_2_, *R*_3_, and *R*_4_) that form a Wheatstone bridge were considered in this study to measure the produced torque on a bicycle’s right crank arm, as shown in Figure 4a. The voltage difference between points A and C is the input voltage (*V_in_*) and that between points B and D is the output voltage (*V_out_*). The voltage of points AD and AB can be described by Equation (2):

(2) $$V_{AD}=V_{in}\frac{R_{1}}{R_{1}+R_{2}}\;\mathrm{and}\;V_{AB}=V_{in}\frac{R_{4}}{R_{3}+R_{4}}$$

The voltage between points AD and AB can be described by Equation (3):

(3) $$V_{out}=\;V_{AD}-V_{AB}=\left(\frac{R_{1}}{R_{1}+R_{2}}\;-\frac{R_{4}}{R_{3}+R_{4}}\right)\;V_{in}$$

The output voltage (*V_out_*) of a Wheatstone bridge is zero when:

(I) $R_{1}R_{3}=R_{2}R_{4}$ and
(II) all resistance have the same value (*i.e.*, $R_{1}=R_{2}=R_{3}=R_{4}$).

A crankset SRM aluminum to fit the Shimano Dura Ace Track FC7710 was selected in this experiment. This crankset is used in track cycling bikes. The V-shaped strain gauge of 1400 Ω ± 0.3% and gauge factor of 2.07 ± 1%, which matches the aluminum installation [37], was installed on the crankset. The V-shaped strain gauge was adopted because it is appropriate for torque measurements. The high-resistance strain gauge was selected because (I) current consumption is reduced, so less produced heat dissipates in the strain gauge; (II) to obtain more alteration of the gauge resistance value, which leads to a relatively wide output voltage range with respect to produced torque; and (III) it is less sensitive when soldering a connection to the amplifier. However, it has a disadvantage, namely, it is more sensitive if signal noise is received. The output of the Wheatstone bridge strain gauge was amplified by the instrumentation amplifier AD 623 with amplifier gain at 46 dB. The output voltage was offset or sifted by *V_supply_*_/2_ to prepare the amplifier output voltage within the microcontroller range. The output of the amplifier was logged by the 10-bit A/D converter, which is embedded in the Atmega 328p microcontroller. The position of the strain gauges is critical to obtain more precise measurements. Therefore, the position was selected by conducting several experiments and taking advantage of the study on the finite element analysis for perpendicular loads presented in [38]. The strain gauges are located between the chain ring and the crank pedal, where the strain gauges are configured as a Wheatstone bridge circuit. The strain gauge positioned on the crank arm was selected in points that achieve a maximum alteration of the strain gauge Wheatstone bridge output voltage. Epoxy RS 473-461 was used to glue the four strain gauges onto a bicycle crank arm on both sides: top (*i.e.*, *R*_1_ and *R*_3_) and bottom (*i.e.*, *R*_2_ and *R*_4_), as shown in Figure 4b,c, respectively.

### 4.2. Wireless Protocols

#### 4.2.1. ZigBee Wireless Protocol

**Figure 5:**
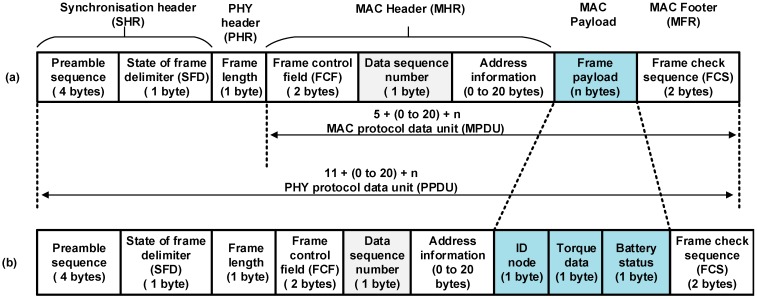
The data packet of IEEE 802.15.4: (**a**) data packet 127 bytes; (**b**) data packet adopted in this experiment 272 bits.

In this paper, a simple ZigBee (XBee S2) network with the sensor, router, and coordinator nodes was established. In our application, the transmission distance between the sensor nodes and coordinator node starts from 32 to 65 m width and length, respectively, of the track cycling field (*i.e.*, velodrome). These distances are measured from the centre of the track cycling area, which has a circumference of 333 m [39]. That means these ranges lie within the XBee S2 communication range for outdoor line-of-sight, as mentioned in the data sheet [40]. As each XBee S2 module is equipped with a USB transmission port, users can connect the XBee S2 module to a computer, which facilitates the configuration parameters. The X-CTU software [41] is used to configure and control the XBee module. However, each XBee S2 module has external digital input/output, analogue-to-digital converters (ADC), data transmit (Tx), and data received (Rx) pins. In addition, there is an external hardware interrupt hibernate pin for sleep purposes [40]. The data packet structure of the XBee S2, which based on IEEE 802.15.4, consists of 127 bytes maximum [42], as shown in Figure 5a. The data structure of the payload of the torque data packet adopted in this experiment consists of three bytes: (I) identification (ID) node of the data torque (one byte); (II) torque data (one byte); and (III) battery voltage sensor node status (one byte), as shown in Figure 5b.

#### 4.2.2. ANT Wireless Protocol

**Figure 6:**
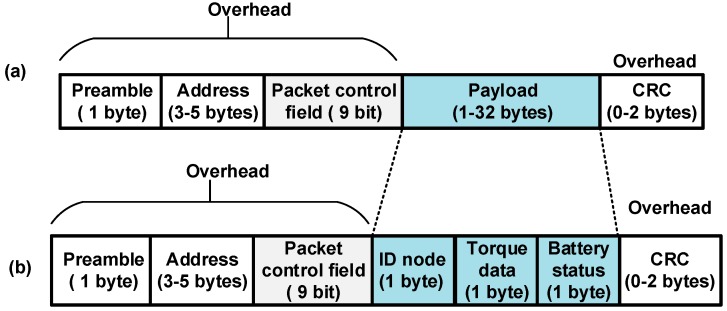
The data packet of ANT RF transceiver: (**a**) enhanced Shockburst; (**b**) enhanced Shockburst adopted in this experiment 97 bits.

In this experiment, the nRF24L01 transceiver, which is produced by Nordic Semiconductor [43], was used to transfer the measured torque data. This transceiver is enhanced with a ShockBurst hardware protocol accelerator and high-speed SPI interface (*i.e.*, 10 Mbps) with a microcontroller. The data packet structure of the ANT, which is shown in Figure 6a, consists of a preamble (1 byte), address of user defined (3–5 bytes it contains the receiver address), packet control bits (nine bits, two bits are used, which are incremented for each new payload, and seven bits are reserved for future products), variable payload length (1–32 bytes of actual data to be sent, depending on the user application) and configurable cyclic redundancy check (CRC, 0–2 bytes, optional). If one byte is used, the polynomial is X^8^ + X^2^ + X + 1, whereas the polynomial is X^16^ + X^12^ + X^5^ + 1 for two bytes. The payload of the torque data packet adopted in this experiment consists of three bytes: (I) ID node of the data torque (one byte); (II) torque data (one byte); and (III) battery voltage status of the sensor node (one byte), as shown in Figure 6b.

**Figure 7:**
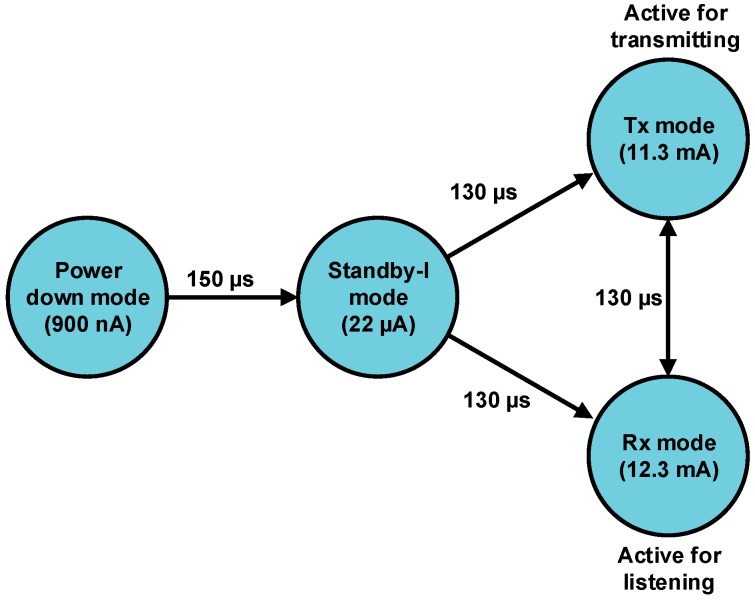
Transition time and current consumption for each mode of ANT.

Figure 7 shows the transition time with the current consumption of the ANT for each mode of operation. To change state from power down mode to Tx/Rx mode or *vice versa*, it must pass through the standby mode.

### 4.3. Power Consumption Algorithm

**Figure 8:**
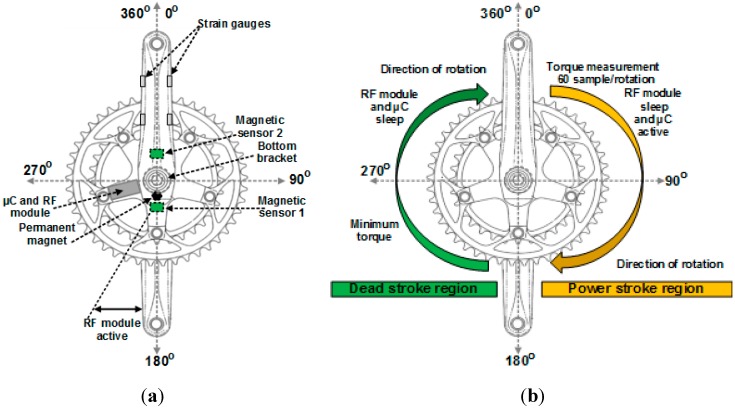
Torque sensor node components installed on the bicycle chainring (**a**) sensors scheme and (**b**) functionality scheme.

A proposed *sleep/wake* algorithm, implemented, which aims to reduce the power consumption of the torque sensor node as well as to measure the crank arm torque. Two magnetic sensors are fixed on the back side of the chainring. Sensor 1 was attached to the bottom of the chainring (*i.e.*, at 180°), and sensor 2 was fixed to the top of the chainring (*i.e.*, 0°). In addition, a permanent magnet was attached to the bicycle bottom bracket, as shown in Figure 8a (sensors scheme). The operation of the *sleep/wake* algorithm can be explained in detail as follows: when the cyclist starts to pedal, for example, the position starts from 0°, as shown in Figure 8b (functionality scheme). The magnetic sensor 1 will pass through the permanent magnet, and it generates a logic HIGH signal that interrupts the microcontroller on pin 2 (INT0). The microcontroller wakes up and enters active mode to start the torque measurements based on Equation (18). The torque data aggregation performed in this experiment is in the form of average values. This approach is used to (I) obtain accurate measurements from more data samples and (II) save a considerable amount of energy by reducing the communication, where the torque data are sampled 60 times and transmitted one time every crank arm rotation. The torque is sampled 60 times for the first half cycle of the chain ring (*i.e.*, 0°–180° also, this phase is named “power stroke”). The torque data were averaged over a power stroke phase and stored in a particular variable. Once, magnetic sensor 2 passes through the permanent magnet; a logic HIGH will be sent from magnetic sensor 2 to pin 3 of the microcontroller. In this case, the first job of the microcontroller sends a control logic LOW signal to wake up the XBee S2 module (where it slept in the power stroke region of the chain ring). Then, the microcontroller generates a small time delay of 10 ms for the purpose of initialising the XBee S2 module from sleep to waking up (note that this time delay becomes 280 µs for the ANT). Later, the microcontroller sends the stored average torque data to the XBee S2 in the form of the data frame. The data frame structure consists of three bytes ID byte, torque data byte and battery status byte. The XBee S2 module wirelessly transmits the torque data to the router node RN (which is installed under the post seat of the rider) in the form of the data packet. After the completion of the transmission process, the microcontroller sends a control logic HIGH signal to the hibernate pin (pin 9) of the XBee S2 to bring it into sleep mode again. At the same time, the microcontroller enters sleep mode during the second half cycle of the chain ring (*i.e.*, 180°–360° also, this phase is named “dead stroke”). Once one rotation is complete, magnetic sensor 1 will again pass through the permanent magnet, and the sequence of the operation is repeated.

For the ANT module, the same algorithm was applied, but the microcontroller does not need to send an external hardware control signal to put the ANT module in wake up or sleep mode, because the ANT module can be brought to power-down mode (*i.e.*, sleep mode) by the program when there are no data ready for transmission. The microcontroller is active only in the power stroke phase to achieve its task and sleep in the dead stroke phase. The RF module transmits the torque data for a fraction of the time when it wakes up and enters into sleep mode most of the time, which leads to more energy being saved. Table 1 shows the state of the microcontroller and RF module at each half cycle relative to the case of two magnetic sensors.

**Table 1:** State of RF module and microcontroller with the case of each magnetic sensor.

Magnetic Sensor 1	Magnetic Sensor 2	Microcontroller State	RF Module State	Degree of Rotation
**1** *	0 ^!^	Wake up	Sleep	0°–180° power stroke phase
**0**	1	Send data and enter sleep	Wake up for a fraction of the time, transmit data and then enter sleep mode	180°–360° dead stroke phase
**0**	0	Depend on the previous case	Sleep	0°–175°, 185°–360°

* High when the magnetic sensor is passing through the permanent magnet. ! Low when the magnetic sensor away from the permanent magnet.

The torque measurement system on the chain ring is divided into two regions: (I) *power stroke region* and (II) *dead stroke region*. Several studies [44,45,46,47,48,49] have proven that the generated torque or applied force on the crank arm in the dead stroke is small compared with the generated torque in the power stroke, *i.e.*, power stroke region is the most effective region that can produce maximum torque. Therefore, the torque measurement in this study focused on the power stroke region and neglected the dead stroke region. This strategy was adopted to reduce the power consumption of the microcontroller to 50%, thereby prolonging the battery life, which is considered a challenge in track cycling application. Prolonging the battery life is especially important when the bicycle is on the track and the battery cannot be replaced or recharged.

### 4.4. Wireless Protocols Current Consumption and Transmission Time Modelling

In this application, the data are transmitted from the sensor node to the router node at event-driven only as a simplex communication. Therefore, no polling and acknowledgement take place at the router node. In this case, the average current consumption *I_avg_* for RF module of the torque sensor node can be computed from Equation (4):

(4) $$I_{avg}=\;\frac{I_{active}t_{active}\;+\;I_{sleep}\left(T_{total}-t_{active}\right)\;}{T_{total}}$$

where *I_active_*, *t_active_*, *I_sleep_*, *t_sleep_* are the RF module current consumption and transmission time in active and sleep modes, respectively, *T_total_* is the time for one crank arm rotation, and *T_total_* − *t_active_* is the sleep time of the RF module.

Rearranging Equation (4), yields Equation (5):

(5) $$I_{avg}=\;\frac{t_{active}}{T_{total}}\;I_{active}+\left(1-\frac{t_{active}}{T_{total}}\;\right)\;I_{sleep}$$

where ($\frac{t_{active}}{T_{total}}$) represent the duty cycle of the RF module, where it is a variable depending on *T_total_* and *t_active_*. *T_total_* relates to the time elapse for one crank arm rotation. The term *t_active_* is an important parameter, which depends mainly on the transmitted RF data packet length. The data packet length is constant in this experiment 272 and 97 bits for XBee S2 and ANT, respectively, as seen in Figure 5b and Figure 6b. The RF transmission time significantly affects the performance of power consumption of a wireless sensor node. The amount of energy of the power source can be reduced by using shorter packet length, which will help maximise the battery life time. Therefore, the transmission time is very important to trade-off between RF wireless standard technologies.

The ZigBee active time *t_active_* (*i.e.*, active transmission time) can be expressed as in Equation (6) [50]:

(6) $$t_{active}=t_{st}+\frac{L_{p}}{R}$$

where *t_st_* is the sleep to active transient time and is equal to 10.2 ms when pin hibernate is used to bring the XBee module to sleep mode [40] as in this experiment, *L_p_* is the packet length in bytes (*i.e.*, 272 bits adopted in this research), and *R* is the XBee S2 data rate of 250 kbps for a 2.4 GHz XBee S2 module. Therefore, the XBee S2 active time for this analysis based on Equation (6) will be 11.288 ms.

Likewise, the ANT enhanced ShockBurst active time *t_active_ESB_* can be expressed as follows [51]:

(7) $$t_{active\_ESB}=T_{UL}+T_{OA}+T_{ACK}+T_{IRQ}+2\;T_{sleep\;to\;active}$$

where *T_UL_* is the upload time to transfer date from microcontroller to ANT through SPI, *T_OA_* is the time on-air, *T_ACK_* is the time on-air ACK, *T_IRQ_* is the interrupt time happening in each autonomous sequence/mode end, and 2*T_sleep to active_* is the transient time from sleep mode to active mode and get back to sleep mode.

The first three parameters of Equation (7) can be expressed as:

(8) $$T_{UL}=\frac{L_{pl}}{R_{SPI}},\;T_{OA}=\frac{L_{p}}{R},\;T_{ACK}=\frac{L_{p}}{R}$$

where *L_pl_* is the payload length in bytes and *R_SPI_* is the data rate of the SPI interfacing between the microcontroller and ANT module.

Therefore, Equation (7) will become:

(9) $$t_{active\_ESB}=\frac{L_{pl}}{R_{SPI}}+2\frac{L_{p}}{R}+T_{IRQ}+2\;T_{sleep\;to\;active}$$

The values of the parameters in Equation (9) as follows; the payload *L_pl_* are three bytes (*i.e.*, 3 × 8 bits/byte = 24 bits), the packet length *L_p_* is 11 bytes plus 9-bit control field (*i.e.*, 11 × 8 bits/byte + 9 = 97 bits) as seen in Figure 6b, the SPI data rate is 10 Mbps, the ANT data rate is 1 Mbps, *T_IRQ_* is 8.2 and 6 µs for data rates of 1 and 2 Mbps, respectively, and *T_sleep to active_* is 280 µs. Therefore, the ANT enhanced shock burst active time *t_active_ESB_* is equal to 764.6 µs. The communication mode and data rate must be pre-set in the configuration process of the RF module. The battery lifetime *L_time_* in hours, which has capacity *C* (in mAh), can be instantly derived from *I_avg_* as in Equation (10):

(10) $$L_{time}=\frac{C\;\left(mAh\right)}{I_{avg}\left(mA\right)}$$

### 4.5. Sensor Node Current Consumption Measurements

The power consumers in the torque sensor nodes are: four strain gauges form a Wheatstone bridge circuit, each strain gauge has a value of 1400 Ω, instrumentation amplifier circuit AD 623, two magnetic sensors, which are used to control the operation of the *sleep/wake* algorithm, stands alone microcontroller Atmega 328p, RF transceiver XBee S2 or ANT.

The measured active current consumption of the strain gauges and instrumentation amplifier were noted as 2.5 and 0.5 mA, respectively. The supply voltage of these two components can be taken directly from any pin of the microcontroller (each pin can drive 40 mA [52]). The microcontroller provided the power to these two parts in power stroke phase and disconnected the power when the crank arm in dead stroke phase. In other words, the two components worked only in the power stroke phase. Therefore, the average current consumption will reduce to 1.25 and 0.25 mA for strain gauges and instrumentation amplifier, respectively. This procedure also is very useful for minimising the current use when the bicycle in standby case. 

The magnetic sensor consumes 0.4 mA when it is active (*i.e.*, closed position) and zero otherwise. In this application, there are two magnetic sensors was used to control the operation of the *sleep/wake* algorithm. Therefore, the current consumption is doubled to 0.8 mA. The sensing angle of each magnetic sensor was measured using the accelerometer sensor. The angle was found in two locations 20° (10° in the position of 0° and 10° in the position of 180°) between the magnetic sensor and the permanent magnet. Consequently, the percentage sensing for one crank arm rotation time is 5% (*i.e.*, 20°/360° × 100%). The magnetic sensor consumes 0.4 mA at this rate and has a zero current consumption the remainder of the time. This leads to the average current consumption of the two magnetic sensors being approximately 0.08 mA, as we will see in Subsection 5.4 (*i.e.*, bar chart of the average current consumption of each component in the torque sensor node).

**Figure 9:**
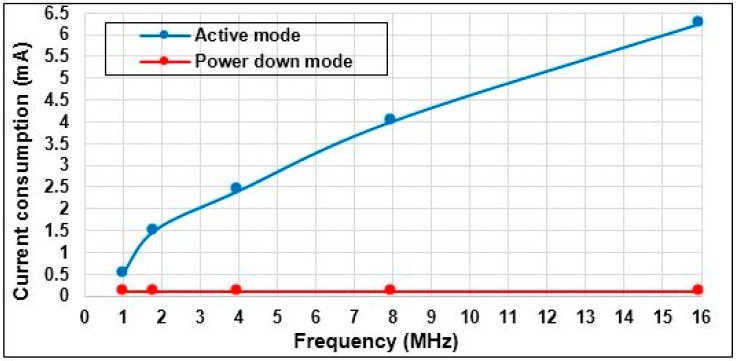
Atmega 328p: current consumption *vs.* operating frequency.

The microcontroller Atmega 328p was implemented in the stand alone circuit to reduce the power consumption of the sensor node. As well as, the choice of the value of the oscillator frequency has played an important part in the energy consumption. Figure 9 based on our measurements, which introduce the relationship between the oscillator frequency of the microcontroller Atmega 328p and current consumption. The figure shows that the oscillator frequency 1 MHz is more appropriate in terms of power consumption. As the microcontroller consumes only 0.5 mA in active mode, this value is matched with Atmega 328 data sheet [52] and 0.09 mA in power down mode. Therefore, this value of the crystal oscillator was adopted in this experiment. In this application, the microcontroller stays in active mode in the power stroke phase (*i.e.*, 0°–180°), and it sleeps in dead stroke phase (*i.e.*, 180°–360°). As a result, the average current consumption of the microcontroller Atmega 328p is 0.295 mA.

In WSN, significant power consumption is consumed by transmission and reception of data [53]. Therefore, the current consumption measurement of active and sleep modes for both ANT and XBee S2 modules was performed based on oscilloscope TBS1000B as follows:

#### 4.5.1. ANT Wireless Protocol Current Consumption Measurements

**Figure 10:**
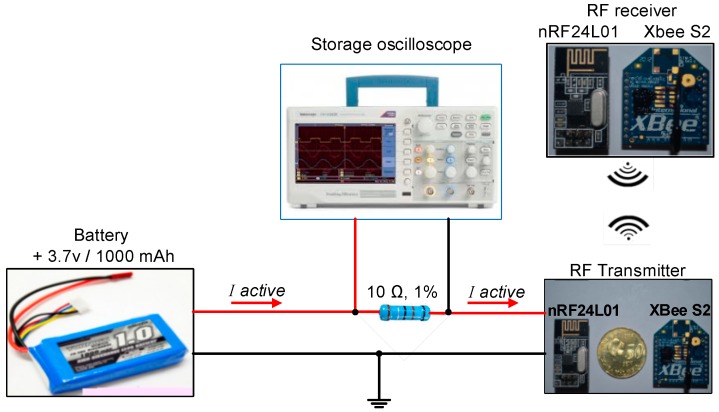
Experimental configuration measurements of current consumption for ANT module.

The active current consumption of the ANT module can be measured using a TBS1000B digital storage oscilloscope as shown in the block diagram in Figure 10. 

**Figure 11:**
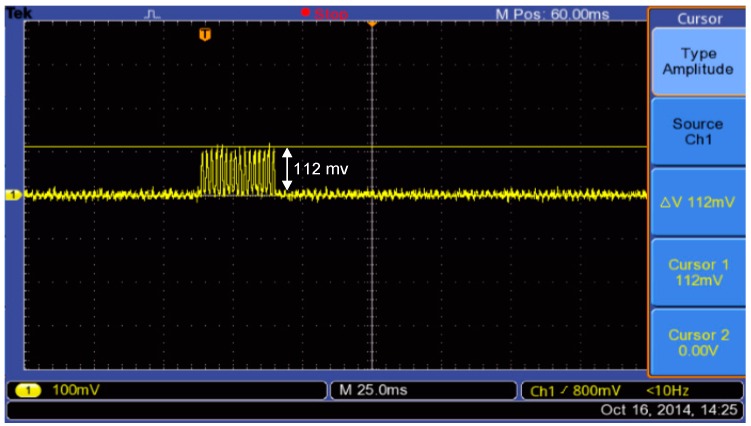
Active current consumption of ANT module during transmission torque data of the bicycle.

The current consumption was measured during the transmission process, when the ANT sensor node communicates with the ANT router node. To measure the current consumed by the ANT wireless module, a shunt resistance of known value is placed between the battery of the sensor node (LiPo rechargeable battery 3.7 V/1000 mAh) and the supply pin of the ANT module. The shunt resistance is 10 Ω with tolerance 1% is selected to minimise the voltage drop in the supply line of the ANT module. Therefore, the current consumed by ANT module can be measured directly by the oscilloscope, this is due to I = V/R, where, the resistance is 10 Ω so I = V/10. Thus, the displayed value on the LCD of the oscilloscope represents the measured voltage across the shunt resistor in millivolts, and the current consumption can be obtained in the order of mA. The voltage across shunt resistance has not to be amplified because, the storage oscilloscope has high sensitivity range 2 mV/div to 5 V/div [54]. Therefore, the peak current consumption of ANT module can be measured directly using the storage oscilloscope. The oscilloscope can capture and store the measured values of current consumption during transmission of torque data as shown in Figure 11. The figure shows that the active peak current consumption is 112 mv/10 Ω = 11.2 mA. The enhanced shock burst active time (transmission time) is mathematically computed based on Equation (9); it is found at 764.6 µs. The current consumption of the ANT module during sleep mode is tiny, and the oscilloscope cannot trace it. Also, an amplifier circuit cannot be used to amplify the sleep current to capture it, that one for minimising the complexity of the system design. Thus, the measurement of sleep current is measured based on a digital multimeter, it is found to be 1 µA.

#### 4.5.2. XBee S2 Wireless Protocol Current Consumption Measurements 

**Figure 12:**
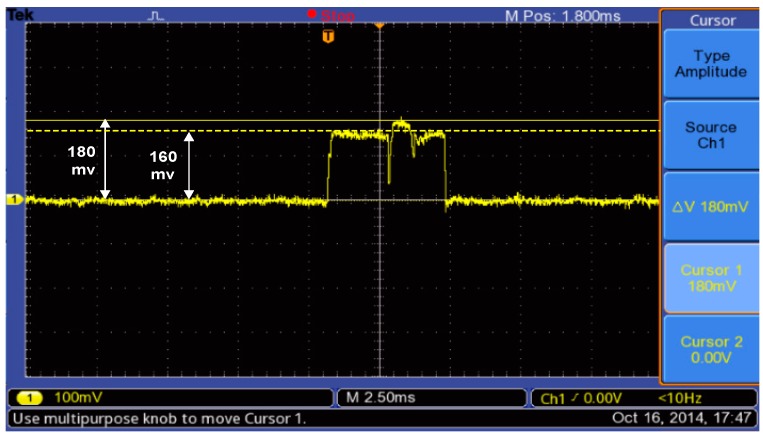
Active current consumption of XBee series 2 module during transmission torque data of the bicycle.

The active current consumption of the XBee S2 module can be measured using the same circuit which shown in block diagram in Figure 10. However, by replacing the ANT module with an XBee S2 module, the storage oscilloscope can capture and store the current consumption of the XBee S2 module as shown in Figure 12 during the transmission process of torque data. Figure 12 displays the active peak current consumption is (180 + 160) mv/2/10 Ω = 17 mA and transmission time is mathematically computed based on Equation (6); it is found at 11.288 ms. 

For the same reason mentioned for ANT module, the current consumption of the XBee S2 module during sleep mode cannot be measured with an oscilloscope. Therefore, the sleep current is measured based on digital Multimeter; it is found at 560 µA.

### 4.6. Mathematical Analytical Model

In this section, a mathematical model for torque sensor node current consumption and bicycle cadence will be derived based on the fundamental laws of physics. This model will facilitate the estimation of the current consumption of the used RF module in the torque sensor node.

From the basics of kinematics, the relationship between the angle (θ) in radians, radius of a circle (*r*), and arc length (*s*) is given [55]:

(11) s=rθ

Because the arc length of a complete cycle of a circle is 2π*r*, Equation (11) will be:

(12) 2πr=rθ then, 2π=θ

The angular velocity ω is defined as the rate of change of angular displacement θ with respect to the change of time *∆t*. The angular velocity of a circle rotating about a fixed axis at a constant speed is:

(13) ω=θ/∆t then, ω=2π/∆t

The angular velocity of the crank set of the bicycle is described as the “cadence (*CAD*)”, or “pedalling rate”. Therefore, the chain ring angular velocity in radian per second is the same as the bicycle cadence (*CAD*) and can be expressed as follows:

(14) ω=CAD=2π/∆t

So, the change of time of one crank arm rotation can be expressed as follows:

(15) ∆t=2π/CAD

Because *∆t* represents the time for one crank arm rotation and *T_total_* in Equation (5) also is the total time for one crank arm rotation. *∆t* will be equal to *T_total_* and a relationship between torque sensor node average current consumption *I_avg_* and bicycle cadence *CAD* can be derived by substituting Equation (15) into Equation (5). In this case, a new mathematical model can be yielded as in Equation (16):

(16) $$I_{avg}=\frac{CAD}{2\pi \times 10^{3}}t_{active}\;I_{active}+\left(1-\frac{CAD}{2\pi \times 10^{3}}t_{active}\;\right)\;I_{sleep}$$

where the units of *CAD* in rad/s, *I_active_*, and *I_sleep_* in mA, *t_active_* in ms, and 10^3^ is the unit transformation from second to millisecond.

The above equation shows a linear relationship between average current consumption and the crank arm cadence. Therefore, the average current consumption of the torque sensor node significantly depends on bicycle cadence. The *t_active_* in Equation (16) is mathematically computed using Equations (6) and (9) for XBee S2 and ANT modules, respectively, in which *t_active_* mainly depends on transmission packet length.

## 5. Results and Discussion

In this section, the results are divided into four phases. The first phase shows the torque calibration process and finds a relationship between the strain gauge output voltage after amplification and produced torque. The torque was calculated based on the applied force on the crank arm and crank arm length, where its length is 170 mm for our bike model. The second phase shows the behaviour of the bicycle crank arm torque and the current consumption of the torque sensor node components based on MATLAB simulation. The third phase aims to select the most appropriate wireless protocol in terms of power consumption. Therefore, a comparison of current consumption and power savings between XBee S2 and the ANT protocol was performed based on experimental measurements, the proposed *sleep/wake* algorithm, and the derived mathematical model. The fourth phase is to estimate the average current consumption of all components in the torque sensor node. In addition, the battery lifetime was estimated based on peak and average current consumption measurement, the proposed *sleep/wake* algorithm, and the derived analytical model.

### 5.1. Torque Calibration

**Figure 13:**
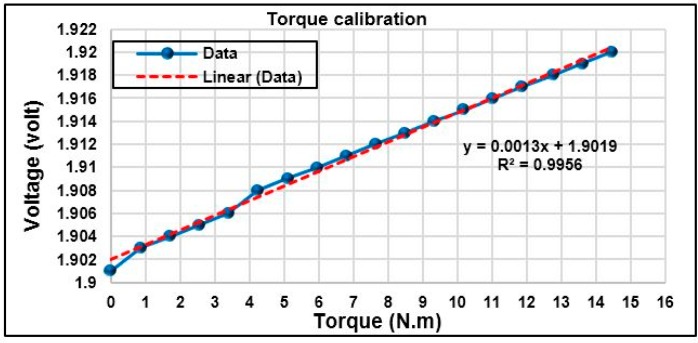
The relationship between the strain gauges output voltage and torque produced in the crank arm.

A calibration process is required to find a relationship between the strain gauge output voltage and the force applied on the bicycle crank arm. A static calibration was performed by suspending known variable weights on the end of the bicycle crank arm. The calibration process has been conducted with known weights (dependent variable) suspended in the crank arm varying from 5 to 85 N with increment steps of 5 N. The amplified voltage (independent variable) of the strain gauge sensor was recorded based on the digital multimeter. When the suspending weight increases during the calibration process, the strain gauge output voltage also increases linearly. Therefore, the weight of 85 N is sufficient to determine a mathematical relationship between the produced torque and the output voltage. This mathematical equation can be utilized to measure the generated torque on the crank arm during cycling when the cyclist is on the track. In addition, the mathematical equation can be used to measure the generated torque out of the calibration range. The relationship between produced torque and strain gauge output voltage is recorded and plotted as shown in Figure 13. This figure displays the linear relationship; therefore, an approximate linear fit line is plotted through data points for determining the relationship between torque on *x- axis* and output voltage on the *y-axis* as in Equation (17):

(17) $$U_{out}=1.3\times 10^{-3}\;T+1.9019$$

where *U_out_* is the instrumentation amplifier output voltage in volts, *T* is the torque produced by the crank arm in (N.m), and 1.9019 is the offset voltage in volts.

Rearrange Equation (17), and Equation (18) can be yielded for torque measurement as follows:

(18) $$T=769.23\;\left(U_{out}-1.909\right)$$

Equation (18) can be fed into the microcontroller algorithm to measure the produced torque on the bicycle crank arm while pedalling.

### 5.2. Simulation Results

**Figure 14:**
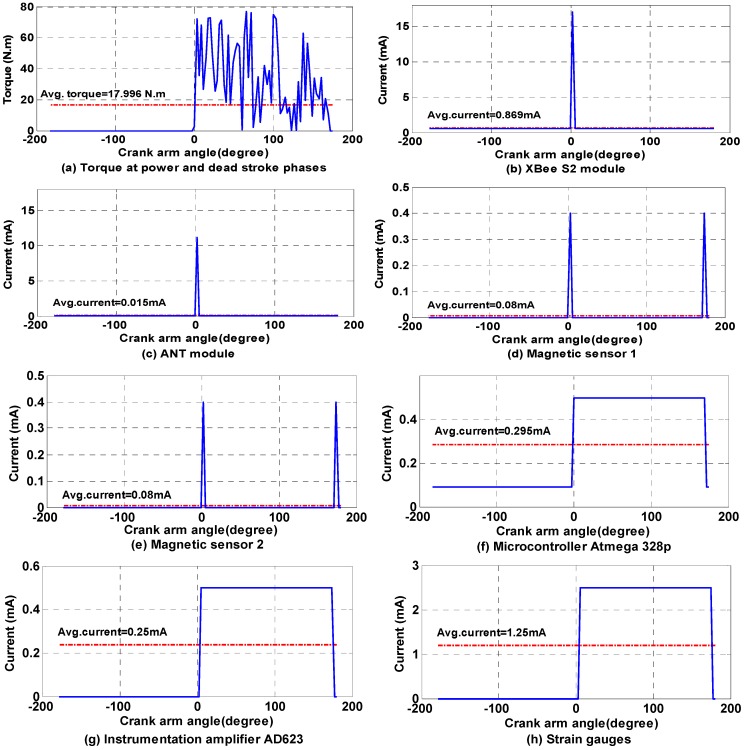
Simulation results for: (**a**) torque values of power and dead stroke phases and average torque every crank arm rotation, and the peak and average current consumption of; (**b**) XBee S2 module; (**c**) ANT module; (**d**) microcontroller Atmega 328p; (**e**) Magnetic sensor 1; (**f**) magnetic sensor 2; (**g**) instrumentation amplifier AD623; and (**h**) strain gauge sensors.

The behaviour of bicycle crank arm torque in two phases (*i.e.*, power and dead stroke; as shown in Figure 8) and the current consumption of each component of the torque sensor node can be simulated. The bicycle crank arm system was simulated based on MATLAB simulation. The simulation was performed with one crank arm rotates 360° (−180°–0°–180°) at a pedalling rate of 100 RPM as shown in Figure 14. This pedalling rate value was selected because it is preferred by experienced cyclists [45], and the professional riders improved their gross efficiency at this pedalling rate [56]. In addition, during the racing tournament, the cyclists held a cadence between 80 and 100 RPM [57]. Figure 14a shows that the torque is zero in dead stroke phase (*i.e.*, −180°–0°), and it has a distinct value in power stroke phase (*i.e.*, 0°–180°) at different crank angles. This situation is due to the application of the *sleep/wake* algorithm, which is more energy efficient.

Moreover, the figure shows the peak and average current consumption (which is indicated by red dotted lines) for each component of the torque sensor node. Figure 14b–d and e show the XBee S2 module, ANT module, magnetic sensor 1, and magnetic sensor 2, respectively. These components are transmitted their data at a fraction of the time and returned to sleep mode, which will help to save more energy. In addition, the microcontroller Atmega 328p activates in power stroke phase and sleeps or powers down in dead stroke phase to reduce its energy consumption by half, as shown in Figure 14f. The instrumentation amplifier AD623 and strain gauge sensors are shown in Figure 14g,h, respectively. The instrumentation amplifier and strain gauge sensors save half of their power by disconnecting their DC power supply in dead stroke phase and connecting it in power stroke phase.

### 5.3. Comparison among XBee S2 and ANT Wireless Protocols

**Figure 15:**
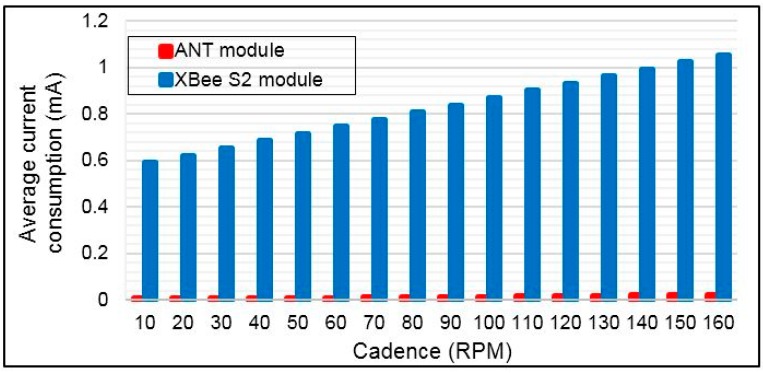
Average current consumption of the ANT and XBee S2 for different bicycle cadence.

The average current consumption for both wireless technology ANT and XBee S2 modules were obtained by applying *sleep/wake* algorithm, as shown in Figure 15. These results are based on measurements of peak current consumption in active and sleep modes for ANT and XBee S2, as seen in Subsection 4.5. The average current consumption for both wireless technologies is computed mathematically based on Equation (16) for different bicycle cadences (CAD) from 10 to 160 RPM. The average speed of track cycling in international competition is 60 km/h [58]. This speed corresponds to the crank arm rotation or cadence of 160 RPM, which is based on our measurements using the SRM system. Therefore, the maximum CAD of 160 RPM is adopted in our current consumption computation. 

**Figure 16:**
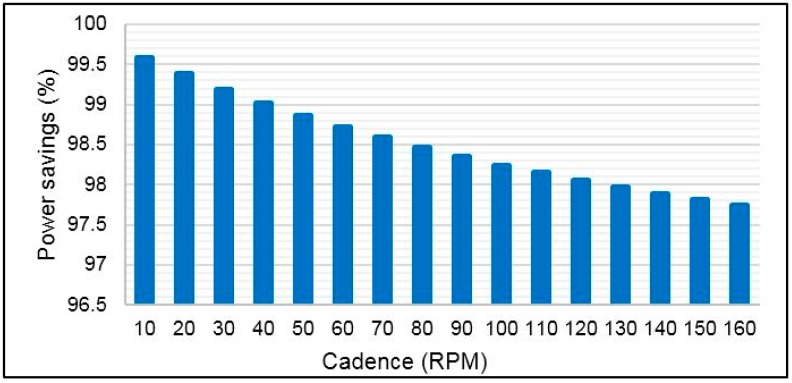
Power saving based on ANT module.

Figure 15 shows that the power consumption of ANT is less significant than XBee S2. The power consumption can be reduced for RF modules when the data rate increases [59], and the data packet time has a short duration [50]. Because the data rate of the ANT is higher than that of XBee S2, the ANT data packet duration is shorter than that of XBee S2. This situation will make the current consumption of ANT for transmitting and receiving packets less than that of XBee S2, as seen in Figure 15. Figure 16 shows significant power savings when the ANT module is used in the torque sensor node. The average power savings are noted to be 98.5% relative to XBee S2. However, the power savings is still high, and between 99.58% and 97.74% is in the cadence range of 10 to 160 RPM.

**Figure 17:**
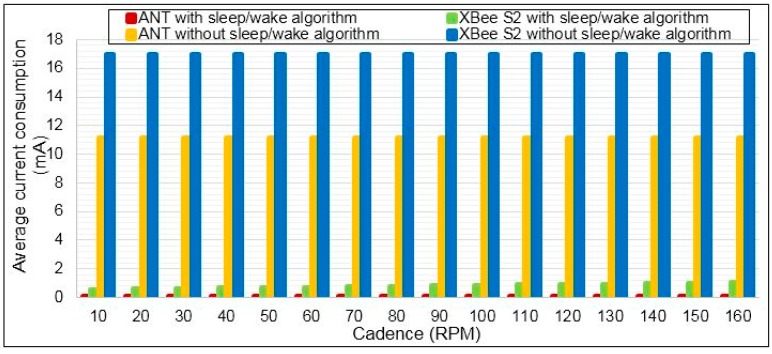
Current consumption comparison between ANT and XBee S2 modules with and without applying *sleep/wake* algorithm.

The current consumption of ANT can be compared with XBee S2 with and without applying the *sleep/wake* algorithm. Once the *sleep/wake* algorithm is applied; the current consumption is dramatically reduced, as shown in Figure 17. This figure shows that the current consumption of both wireless technologies ANT and XBee S2 when applying the *sleep/wake* algorithm is better than without using this algorithm. The average power savings of using XBee S2 based on a *sleep/wake* algorithm are 94.97% relative to XBee S2 without applying the *sleep/wake* algorithm. However, the average power savings of using ANT based on a *sleep/wake* algorithm is 99.87% relative to ANT without using the *sleep/wake* algorithm.

### 5.4. Sensor Node Average Current Consumption and Battery Lifetime

**Figure 18:**
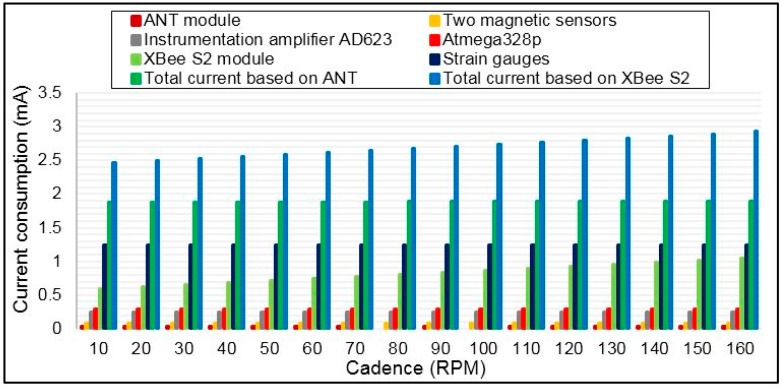
Average current consumption of each component in the torque sensor node as a function of bicycle cadence by applying the *sleep/wake* algorithm.

**Figure 19:**
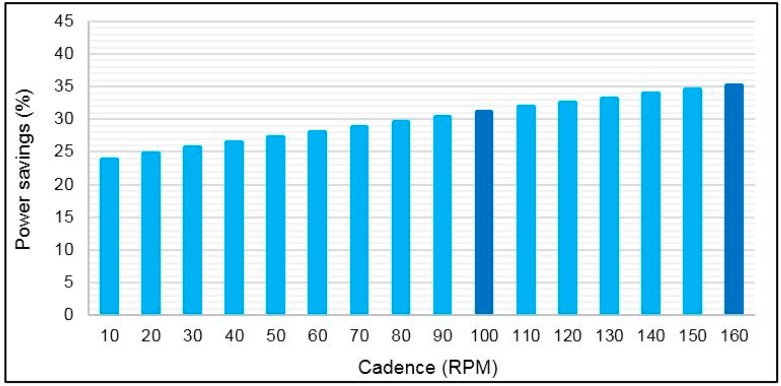
Power saving of the torque sensor node based ANT module.

**Figure 20:**
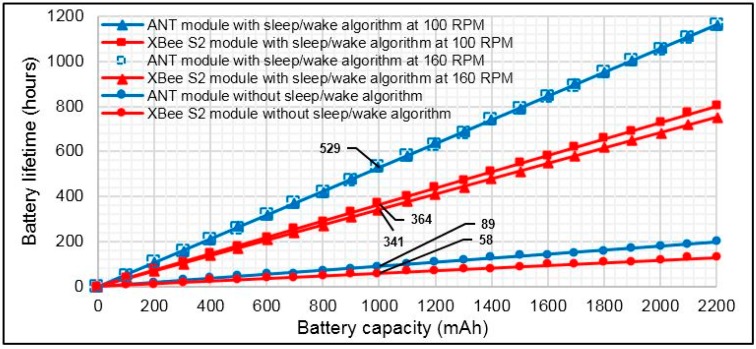
Estimated battery lifetime *vs.* usable battery capacity in the torque sensor node for three cases based on the use of ANT and XBee S2 modules.

The average current consumption of each component and the average total current consumption of the torque sensor node were computed based on current measurements in active and sleep modes and Equation (16). The results are shown in Figure 18 for different bicycle cadences on the basis of the application of the *sleep/wake* algorithm during the crank arm rotation. This figure shows that the most power is dissipated in the strain gauge transducers and XBee S2 module, whereas a small amount of power is consumed by ANT. In contrast, the torque sensor node consumes more power when it uses the XBee S2 module compared with ANT. Figure 19 shows the power savings of the torque sensor node based on ANT relative to XBee S2 for cadence range. This figure shows that the power saving increases with cadence because the total average current consumption of the XBee S2 module increases with cadence, whereas the total average current consumption increases slightly when ANT is used. The obtained power savings are 31% and 35% for cadences of 100 and 160 RPM, respectively. As expected, the power saving in this case is less than in the first case (*i.e.*, the case in Subsection 5.3). This outcome occurred because in the first case, the average current consumption computation was achieved based on RF modules alone, whereas for this case, the computation was performed based on sharing all the components of the sensor node. Figure 20 shows the estimated battery lifetime of the torque sensor node with the used battery capacity for the two wireless technologies for three cases: (I) operation without the *sleep/wake* algorithm (*i.e.*, conventional operation); (II) operation of the *sleep/wake* algorithm at 100 RPM; and (III) operation of the *sleep/wake* algorithm at 160 RPM. The battery lifetime of the torque sensor node was prolonged to 529 h in the second and third cases when the ANT module was used in the sensor node, noting that the battery capacity is 3.7 V/1000 mAh, whereas the battery lifetime was 364 and 341 h for 100 and 160 RPM, respectively when the XBee S2 module is used. The battery life in the first case (*i.e.*, conventional operation) was 89 and 58 h for the ANT and XBee S2 modules, respectively, which is significantly less than that of the second and third cases. The above results indicated that the ANT protocol is more convenient for the torque sensor node in terms of power consumption.

## 6. Conclusions

Minimising the current consumption in the present application is considered to be the primary challenge because the torque sensor node is battery-powered. The XBee S2 and ANT wireless protocols were investigated practically in terms of current consumption, as were other components of the torque sensor node. The sensor node current consumption was reduced by applying a proposed *sleep/wake* algorithm. The results confirmed that the ANT protocol is more suitable for this application than XBee S2 in terms of current consumption and battery lifetime, especially when the *sleep/wake* algorithm is applied. In contrast, the XBee S2 was more appropriate than ANT in terms of communication distance between cyclist and coach. Therefore, a trade-off between power consumption and communication range is necessary.

From this application, it can be observed that the current consumption of the torque sensor node does not depend solely on the RF wireless protocols (*i.e.*, XBee S2 or ANT protocols), but also depends on other components such as strain gauges and instrumentation amplifier AD623, which consume more power than the RF wireless protocol when the *sleep/wake* algorithm is applied. The hardware selection greatly affects the sensor node power consumption. Thus, microcontroller Atmega 328p was implemented as a stand-alone board with a low operating oscillator frequency. The high value of the strain gauge sensor and low power instrumentation amplifier AD623 improved the current consumption of the sensor node. In addition, the magnetic sensor (reed switch) consumes little current when it is ON for a fraction of the time and zero current for the rest of the time. An analytical model was derived to correlate between the torque sensor node average current consumption and the bicycle cadence. The derived mathematical model facilitated the estimation of the average current consumption and battery lifetime of the sensor node at different pedalling rates. Future work will focus on improvement on the accuracy and precision. This goal can be accomplished by sending the torque data in 16-bit. The torque data in the present study are sent only in 8-bit to reduce the XBee S2 packet length, thus reducing the power consumption.
